# Determination of the size distribution of non-spherical nanoparticles by electric birefringence-based methods

**DOI:** 10.1038/s41598-018-27840-0

**Published:** 2018-06-22

**Authors:** Paloma Arenas-Guerrero, Ángel V. Delgado, Kevin J. Donovan, Kenneth Scott, Tommaso Bellini, Francesco Mantegazza, María L. Jiménez

**Affiliations:** 1 Department of Applied Physics, Univ. Granada, Granada, 18071 Spain; 2 School of Physics and Astronomy, Queen Mary Univ. London, London, E14NS UK; 3 Department Med. Biotechnol. and Translat. Med., Univ. Milan, Milan, I20090 Italy; 4 Department Medicina e Chirurgia, Univ. Milano-Bicocca, Vedano al Lambro, MB 20854 Italy

## Abstract

The *in situ* determination of the size distribution of dispersed non-spherical nanoparticles is an essential characterization tool for the investigation and use of colloidal suspensions. In this work, we test a size characterization method based on the measurement of the transient behaviour of the birefringence induced in the dispersions by pulsed electric fields. The specific shape of such relaxations depends on the distribution of the rotational diffusion coefficient of the suspended particles. We analyse the measured transient birefringence with three approaches: the stretched-exponential, Watson-Jennings, and multi-exponential methods. These are applied to six different types of rod-like and planar particles: PTFE rods, goethite needles, single- and double-walled carbon nanotubes, sodium montmorillonite particles and gibbsite platelets. The results are compared to electron microscopy and dynamic light scattering measurements. The methods here considered provide good or excellent results in all cases, proving that the analysis of the transient birefringence is a powerful tool to obtain complete size distributions of non-spherical particles in suspension.

## Introduction

Non-spherical micro and nanoparticles have gained a growing interest because of the number of applications associated specifically to shape (drug delivery, nanosensors, electrorheology, printed electronics, photodetectors,…^1–5^) as well as their possible use as models with well defined aspect ratios. Progress in size characterization techniques for these particles is thus essential both for further development of these applications and for general progress in nanoparticle synthesis^6–8^. While historically most efforts in size characterization have been directed towards spherical geometries^9^, recent works have specifically targeted the size characterization of non-spherical particles.

Thus, techniques such as micro-flow imaging, asymmetrical flow field fractionation, dynamic light scattering (DLS), focused beam reflectance measurements or centrifugal separation analysis have been applied to particles with different shapes, although there are still many limitations^10–14^. Very promising results have been achieved with the analysis of the optical absorption spectra of two-dimensional materials like MoS_2_^15^. For more general non-spherical geometries, the study of the electric birefringence of dispersed particles has proven a useful tool for size characterization^16^.

## DLS for size characterization of non-spherical particles

Dynamic light scattering is an *in situ* size characterization technique for nanoparticles in suspension based on the measurement of their translational diffusion coefficient, related to the length *L* of their major axis as

1 $$D=\frac{{k}_{B}T}{3\pi \eta L}{F}_{D}$$

where *η* is the viscosity of the solvent, *k*_*B*_ the Boltzmann constant and *T* the temperature of the sample. *F*_*D*_ is a geometrical coefficient depending on the shape, but not the size, of the particles. For spheres, *F*_*D*_ = 1. Expressions of *F*_*D*_ for the geometries used in this work can be found in the Supporting Information.

DLS is the most used tool for the size characterization of spherical particles, commercial equipments being available and widespread. In many cases, these commercial devices, prepared for spheres, are used for the size characterization of non-spherical particles in suspension^11,17–23^. However, it has been shown that, for these geometries, the contribution of rotational diffusion, not taken into account, can have important effects on the DLS determinations^24,25^, and hence alternative approaches must be proposed.

In recent years, an effort is being made to develop depolarised dynamic light scattering as a suitable tool for the characterization of non-spherical particles^12,26–31^. Nevertheless, this technique still faces experimental challenges and cannot yet be considered as a widespread method^32^. Moreover, the results are still limited to average dimensions of the particles and can hardly provide complete size distributions.

## Electric birefringence-based methods for size characterization

Electric birefringence (EB) is the macroscopic optical anisotropy that emerges in a suspension of non-spherical particles when they are oriented by the application of an external electric field^33,34^. Upon removal of the field, thermal agitation randomises the orientation of the particles and the birefringence of the sample decays. This randomisation process, controlled by rotational diffusion and strongly size-dependent, can be monitored via the measurement of the birefringence decay and, in this manner, information on the size distribution of the suspended particles can be extracted.

The use of birefringence-based methods for particle size characterization presents several advantages. First of all, this technique analyses the particles directly in suspension, so they do not need to be dried out or placed near a surface. This avoids the corruption of the sample and the modification of the state of aggregation. Secondly, a very wide range of sizes, including the nano and microscale, can be studied with the same method^35–39^. In this technique, it is not necessary to know the concentration of the sample, as long as it can be ensured that particle interactions do not modify the rotational diffusion of the single particle.

EB-based methods measure the rotational diffusion coefficient Θ of the particles, which depends on size as

2 $${\rm{\Theta }}=\frac{3{k}_{B}T}{\pi \eta {L}^{3}}{F}_{{\rm{\Theta }}}$$

where *F*_Θ_ is a shape factor, with a value of 1/3 in the case of spheres. Expressions of *F*_Θ_ for the geometries used in this work can be found in the Supporting Information. Note that the dependence of rotational diffusion on particle size is much stronger than that of translational diffusion. Therefore, EB measurements are more sensitive to size than DLS experiments. Moreover, the EB measurements are not affected by translational diffusion, which simplifies the data analysis as compared to DLS, affected by both rotational and translational diffusion.

With this motivation, in this work we analyse the suitability of three different birefringence-based approaches for particle size determination: the stretched exponential (SE)^40,41^, multi-exponential (ME)^42^ and Watson-Jennings (WJ)^43^ methods. From the analysis of a single birefringence decay, the former enables the determination of the average particle dimensions, and the latter two can provide a complete size distribution of the particles in the sample. It is worthy to note that the ME method was proposed elsewhere^42^, but it has not been experimentally tested for colloidal particles.

These methods are applied to suspensions of four different types of elongated particles, namely teflon rods, goethite needles, and single- and double-walled carbon nanotubes, and two types of planar particles, sodium montmorillonite particles and gibbsite platelets. The obtained results are compared amongst the three methods and to size distributions obtained from electron microscopy (EM) and DLS measurements.

Recall that when a suspension of non-spherical particles is subjected to an external electric field, this will induce an anisotropic distribution of particle orientations because of the torque of the field on the induced and/or permanent dipoles of the particles and their electrical double layers. As a consequence, an optical anisotropy arises at the macroscopic scale, this is, the refractive index of the suspension along the direction parallel to the electric field $({n}_{\parallel })$ is different to that along the perpendicular direction (*n*_⊥_). The difference ${\rm{\Delta }}n={n}_{\parallel }-{n}_{\perp }$ is the electric birefringence of the system.

When the field is turned off, the particles randomise their orientation due to rotational diffusion. The three EB-based methods mentioned for size characterization measure the diffusion coefficient Θ of the particles analysing this transient behaviour of electric birefringence upon removal of the electric field. They can be summarised as follows (more detailed information is provided in the Supplementary Information file):

### SE Method

The decay of the electric birefringence of a polydisperse sample is expected to be, under reasonable assumptions, in the form of a stretched exponential function^40,41^

3 $${\rm{\Delta }}n(t)={\rm{\Delta }}{n}_{0}\,\exp [-{(\frac{t}{\tau })}^{\alpha }]$$

where Δ*n*_0_ is the initial value of the birefringence, *α* a polydispersity factor and *τ* a characteristic decay time, which can be related to the average rotational diffusion coefficient of the particles as ${\rm{\Theta }}=\frac{1}{6\tau }$.

As previously mentioned, the diffusion coefficient is strongly dependent on the particle size (Equation 2), expressions being available for simplified geometries (presented in the Supporting Information). Thus, from the obtained value of Θ, the average dimensions of the particles can be estimated. Note that, since the birefringent signal is proportional to the particle volume, the average values provided by this method are volume-weighted.

### WJ method

The Watson-Jennings method^43^ assumes that the length distribution of the sample has the shape of a log-normal function

4 $$P(L)=\frac{1}{L\sigma {\mathrm{(2}\pi )}^{\mathrm{1/2}}}\exp \{\frac{-{[\mathrm{ln}(L/{L}_{{\rm{M}}})]}^{2}}{2{\sigma }^{2}}\}$$

where *L*_M_ is the median and *σ* the breadth parameter of the distribution. For highly oriented systems, these parameters are directly related to the initial logarithmic derivative, *D*_WJ_, and the area under the curve, *I*_WJ_, of the normalised birefringence decay, as

5 $${D}_{{\rm{WJ}}}=\frac{d}{dt}{(\mathrm{ln}\frac{{\rm{\Delta }}n(t)}{{\rm{\Delta }}{n}_{0}})}_{t\to 0}=-\,\frac{6{L}_{{\rm{M}}}^{3}}{{F}_{{\rm{\Theta }}}}{{\rm{e}}}^{15{\sigma }^{2}\mathrm{/2}}$$

6 $${I}_{{\rm{WJ}}}={\int }_{0}^{\infty }\frac{{\rm{\Delta }}n}{{\rm{\Delta }}{n}_{0}}dt=\frac{{{\rm{e}}}^{9{\sigma }^{2}}}{{D}_{{\rm{WJ}}}}$$

In this manner, *L*_M_ and *σ*, and hence the size distribution of the sample, can be obtained from a single birefringence decay measurement.

### ME method

The multi-exponential method^42^ assumes that the birefringence decay can be built as a superposition of single exponential decay processes, each corresponding to a fraction of a well-defined size, as

7 $$\frac{{\rm{\Delta }}n(t)}{{\rm{\Delta }}{n}_{0}}=\sum _{i=1}^{N}{C}_{i}{e}^{-t/{\tau }_{i}}$$

The coefficients *C*_*i*_ weight the contribution of each population to the overall signal, which in the case of birefringence is proportional to the particle volume. Thus, these coefficients provide a volume distribution of the particle dimensions in the sample. The characteristic decay time for each population is determined from the respective rotational diffusion coefficient with the expression *τ*_*i*_ = 1/6Θ_*i*_, where Θ_*i*_ can be calculated if the geometry of the particle is known. The values of *t* and Δ*n* are obtained from the measured EB decay. The equation system 7 is solved to obtain the *C*_*i*_.

In contrast with the WJ method, no specific form is assumed for the distribution. Moreover, unlike the SE method, this technique provides a complete size distribution of the particles in suspension. In order to minimize the contribution of experimental noise, the birefringence decay data must be smoothed in some manner. In this work, we have chosen to use the fitting to a stretched-exponential function for the application of the ME Method. Furthermore, it must be noted that a reasonable length range must be found to carry out the calculations. For this purpose, we used the size estimation provided by the SE method.

## Methods

We have selected particles both in the micro and nanoscale, with different shapes, including elongated and planar geometries. Goethite and teflon (PTFE) rods were purchased from Sigma Aldrich (Spain) and Ausimont (Italy) respectively. Single- and double-walled carbon nanotubes were acquired from Carbolex (USA) and US Research Nanomaterials (USA). As planar particles we studied gibbsite platelets, synthesised following the procedure described elsewhere^44^, and sodium montmorillonite (NaMt) particles, obtained from bentonite (Serrata de Níjar, Spain) by a process of homoionisation to the Na-form^45^. Briefly, this consisted in rinsing the sample with a 1 M sodium chloride solution, and redispersing in deionised water (Milli-Q Academic Millipore, France). All samples were prepared by simple dispersion of the particles in the solvent with the help of a sonicator.

The electric birefringence data presented in this work have been measured in different laboratories using the standard device, based on the measurement of the changes in light transmission though an optical setup containing the suspension, which is subjected to electric field pulses^46^. The DLS measurements have been performed with a commercial apparatus (Zetasizer, Malvern Instruments (UK)) which provides the diameter distribution of the particles assuming they are spherical. In order to obtain the major length of the non-spherical particles, we recalculate the measured translational diffusion coefficient using the expression for spheres and then obtain *L* from equation 1.

Note that, in order to apply DLS and the three EB-based methods, expressions for the translational and rotational diffusion coefficients of the particles are needed. For this purpose, here we assume that they can be approximated either by short rods, long rods, thin disks or oblate spheroids and use equations 1 and 2, and the expressions of *F*_*D*_ presented in the supporting information, where *ρ* is the aspect ratio, this is, the quotient between the major and minor axis of the particles.

### Data Availability

All data generated or analysed during this study are included in this published article (and its Supplementary Information file).

## Results

**Figure 1:**
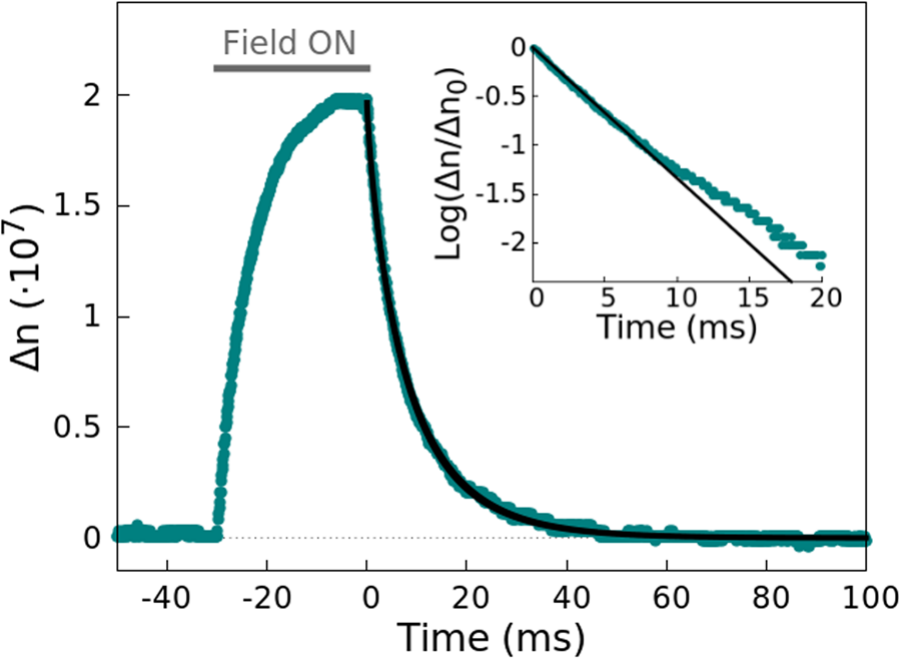
Birefringence signal of the goethite sample subjected to an electric field pulse of 30 ms. The points are the experimental data, and the black line a fitting of the decay to a stretched exponential function. In the inset, the data for short times (points) are presented in logarithmic scale, along with a linear fitting (black line), to show the linear dependence.

Figure 1 shows the birefringence signal of the goethite sample. Here, it can be observed that, when the electric field is turned on, the birefringence grows and reaches a stationary value. When the field is turned off, the birefringence decays to zero following a stretched exponential function. In this figure, we also present this decay in logarithmic scale for short times, in order to calculate the initial logarithmic derivative required for the WJ method (Eq. 5). The birefringence decay of all the studied suspensions can be consulted in the Supporting Information of this contribution. The results found for the six samples with the SE, ME and WJ methods are presented and discussed below, together with the electron microscopy and DLS measurements.

### Goethite needles

**Figure 2:**
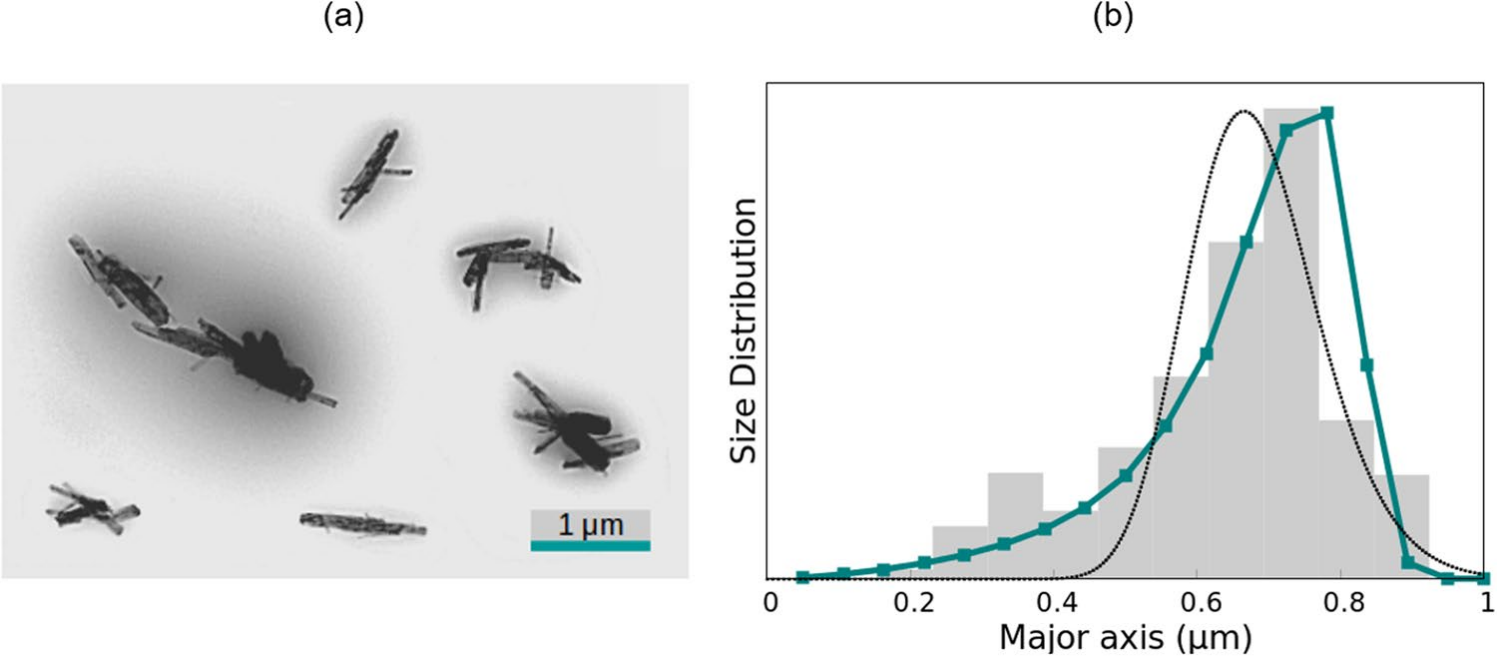
(**a**) Electron microscope image of the goethite particles. (**b**) Volume size distribution obtained via the ME method (coloured squares and line) and WJ method (dotted black line), compared with the distribution determined from electron microscopy (grey bars).

The electric birefringence of goethite particles, purchased from Sigma Aldrich (USA), was analysed in aqueous suspension, at 10 mg/L. The sample was subjected to electric field pulses of 5 V/mm and 100 kHz. These and subsequent experiments with aqueous suspensions were carried out at 15 °C, to minimise heating effects and solvent evaporation. Figure 2a shows a transmission electron microscopy image of these particles, from which the volume-averaged length (0.64 *μ*m) and mean aspect ratio (*ρ* = 12.3) have been obtained. For calculations, we used the expression of the diffusion coefficients of short rods and the microscopy value of *ρ*. The assumption of constant aspect ratio is not expected to produce a significant effect on the results, since the /*rho*-dependence of Θ through *F*(Θ) is very smooth, as compared to the *L*^−3^ dependence. In fact, when contrasted with the assumption of constant diameter, both obtained distributions are comparable, although the constant aspect ratio approximation yields better results (Fig. S2 of the Supplementary Information).

The length distributions provided by the ME and WJ methods, together with the one obtained from EM, are shown in Fig. 2b. The values of the mean length and standard deviation of all methods, including DLS, can be consulted in Table 1. All presented distributions and averages are in volume.

**Table 1:** Volume-averaged length of the major axis of the particles (and standard deviation of the distribution when available) obtained via EM and DLS measurements and with the SE, ME and WJ methods.

	Goethite	PTFE
SE method	0.67 *μ*m	0.50 *μ*m
ME Method	0.67 *μ*m (21%)	0.49 *μ*m (31%)
WJ method	0.68 *μ*m (14%)	0.46 *μ*m (23%)
Microscopy	0.64 *μ*m (24%)	0.50 *μ*m (22%)
DLS	0.99 *μ*m (38%)	0.24 *μ*m (45%)
	**SWNTs**	**DWNTs**
SE method	0.88 *μ*m	1.61 *μ*m
ME method	0.89 *μ*m (53%)	1.69 *μ*m (98%)
WJ method	0.94 *μ*m (28%)	1.60 *μ*m (57%)
Microscopy	0.89 *μ*m (62%)	0.86 *μ*m (50%)
DLS	0.58 *μ*m (45%)	1.10 *μ*m (27%)
	**Gibbsite**	**NaMt**
SE method	0.23 *μ*m	1.74 *μ*m
ME method	0.23 *μ*m (25%)	1.73 *μ*m (42%)
WJ method	0.24 *μ*m (20%)	1.61 *μ*m (33%)
Microscopy	0.25 *μ*m (25%)	1.62 *μ*m (43%)
DLS	0.18 *μ*m (37%)	0.42 *μ*m (35%)

The SE method (*τ* = 7.96 ms and *α* = 0.836) furnishes a mean length of 0.67 *μ*m, within a 5% difference of the microscopy value, and coinciding with the average length provided by the ME method. As shown in Fig. 2b, the ME method yields excellent results, as the obtained distribution is almost identical to the one found from EM. The WJ method also gives satisfactory results, with a mean length of 0.68 *μ*m. However, the WJ distribution is narrower than the EM results and its maximum is slightly shifted to the left.

In Table 1, the discrepancy of the DLS measurement with EM and the SE, ME and WJ methods is evident. The distribution obtained by DLS is wider and strongly shifted to larger lengths than all other results.

### PTFE rods

**Figure 3:**
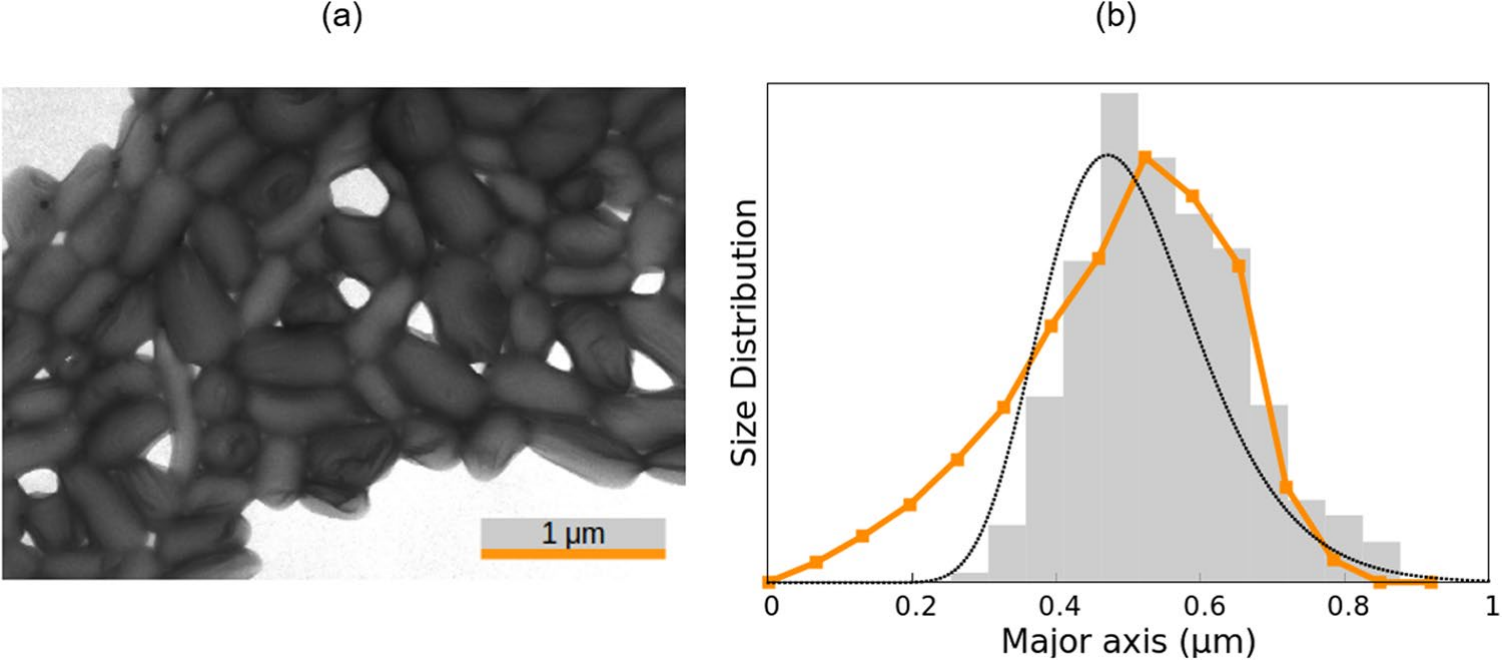
Same as Fig. 2 but for the PTFE rods.

The elongated PTFE particles were purchased from Ausimont (Italy). In the experiments, an electric field of 40 V/mm and 100 kHz was applied to a 0.1% v/v PTFE aqueous suspension. Figure 3a shows a transmission electron microscope image of the PTFE rods, where it can be observed that they present a significant polydispersity. From this picture, values for the volume-averaged length (0.50 *μ*m) and mean aspect ratio (*ρ* = 2.0) were obtained. Again, we modelled the particles as short rods and assumed the microscopy value of *ρ*, the same approximation as in the case of goethite.

The results obtained by all methods are presented in Fig. 3b and Table 1. The SE method (*τ* = 12.00 ms and *α* = 0.721) provides a mean length of 0.50 *μ*m, which is in perfect agreement with both the EM and the ME results. As shown in Fig. 3b, the size distribution found by the ME method is very similar to the microscopy one for lengths over 0.40 *μ*m. However, this method predicts the presence of small particles that were not identified in the image, which results in a slightly broader distribution. Note that the PTFE particles present an aspect ratio of 2, which is in the limit of the application range of the expressions used for the diffusion coefficient of short rods. Hence, the deviations could be related to the geometrical approximation. In spite of this, the difference in the average length is only 3%. The WJ method gives very good results, with a size distribution that is very similar to the EM one, but slightly shifted to the left, which results in a value of the mean length 8% lower than expected.

In the DLS measurement of the PTFE sample, two peaks were observed. The one corresponding to shorter lengths is spurious, due to the rotational diffusion effect on the DLS measurement, as already reported elsewhere^25^ for suspensions of gold nanorods. This peak was not taken into account for the calculation of the average length and standard deviation presented in Table 1. Despite this consideration, the obtained mean length is smaller by 50% than measured by EM and the EB-based methods, and the distribution is notably wider.

### Single-walled carbon nanotubes

Single-walled carbon nanotubes (SWNTs) were suspended 1,2-dichloroethane with a volume fraction of 10^−4^%. The carbon nanotubes required a very high field, 250 V/mm, to be oriented. The field frequency was 1 kHz and the measurement temperature 20 °C.

**Figure 4:**
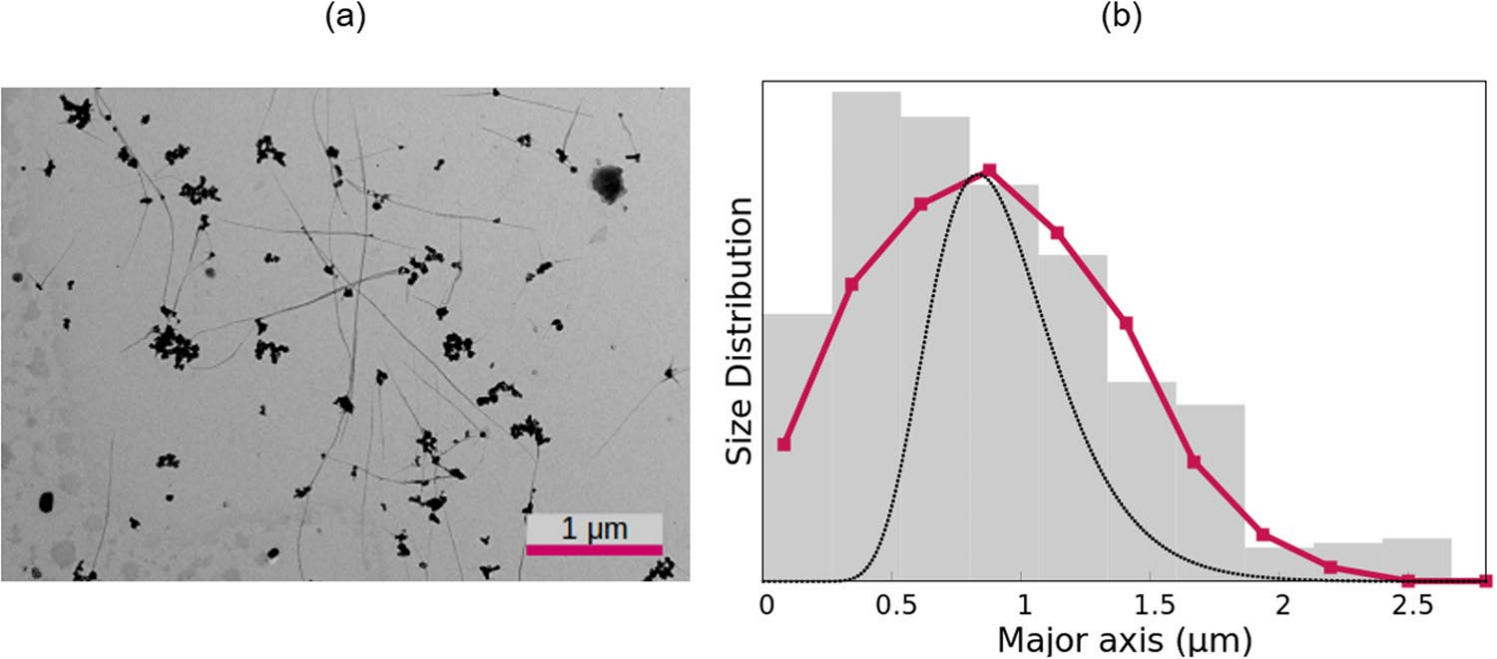
Same as Fig. 2 but for the SWNT suspension.

A transmission electron microscope image of the SWNTs can be found in Fig. 4a, where it can be observed that the particles appear bundled, with a mean bundle width of 12 nm. From 300 particles, an average length value of 0.98 *μ*m was obtained. The polydispersity in length is quite important, whilst the width is more uniform. Naked-eye distinction of the bundles in the picture is not straightforward, but an effort has been made to look at the image at different zooms to account for all present lengths. In calculations, we used the expression of the diffusion coefficients of long rods and assumed a constant particle diameter of 12 nm. All results are presented in Table 1 and Fig. 4b.

The birefringence decay fits perfectly to a stretched exponential function (*τ* = 6.95 ms, *α* = 0.545), and the SE method furnishes a value for the volume-averaged length very similar to that found from EM, with less than a 1% difference. The ME method also provides excellent results, giving a size distribution very similar to the one found from microscopy determinations, the more so taking into account the poor particle distinction in the picture. The WJ method calculates a mean length reasonably similar to the EM result, within a 5% difference, but the distribution is notably different, as it is clear from the comparison of their standard deviations.

The results obtained by the EM and the EB-based methods are also compatible with the mean length value of 1.08 ± 0.20 *μ*m found elsewhere^47^ for a similar sample via the measurement of the electrical polarizability anisotropy of the conducting tubes. On the other hand, the results found by DLS deviate strongly from all other methods, being the given mean length 35% smaller than, for example, the EM value.

### Double-walled carbon nanotubes

**Figure 5:**
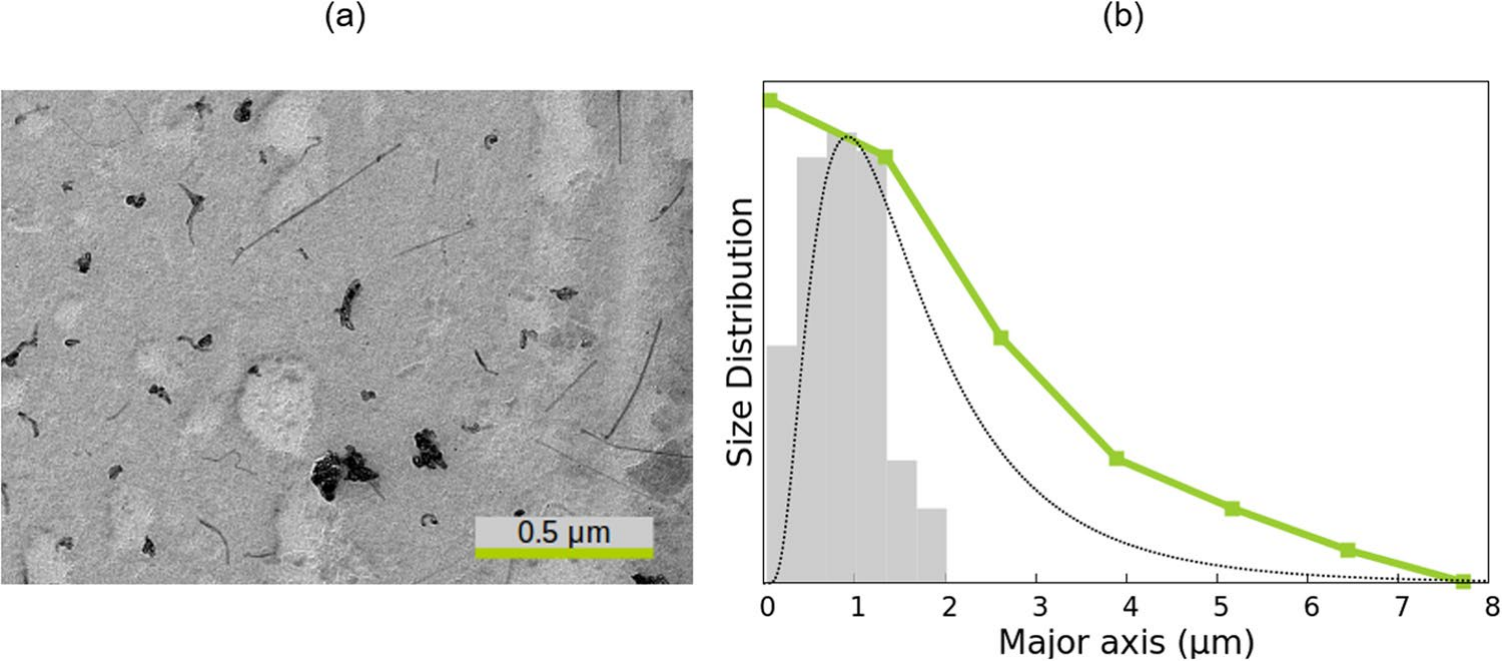
Same as Fig. 2 but for the DWNT sample.

The birefringence of double-walled carbon nanotubes (DWNTs) was determined in the same experimental conditions as the SWNT sample. A transmission electron microscope image of these particles (Fig. 5a) shows that they are also bundled, with a bundle width of 9 nm. From 75 tubes, a mean length of 0.86 *μ*m was obtained. Again, the particles are not easily distinguished. For the calculations, we use a constant width of 9 nm and the diffusion coefficient of long rods.

The birefringence decay of this suspension is quite slow and could be fitted by a stretched exponential function with fitting parameters *τ* = 33.4 ms and *α* = 0.3166, indicating a very polydisperse sample. The results are presented in the usual way in Table 1 and Fig. 5b, where it can be observed that the volume-averaged particle length provided by the three EB-based methods, around 1.63 *μ*m, is quite different from that obtained via EM.

These findings are in line with the results reported elsewhere^47^, where the length of the bundles in a similar sample was obtained from the experimental determination of their electrical polarizability. The obtained mean length, 1.63 ± 0.10 *μ*m, is identical to the value found by our methods. These results suggest that, when in suspension, the DWNTs are more entangled than observed by EM. This is one of the advantages of the birefringence-based methods over the use of microscopy techniques, since particles are analysed directly in suspension and do not need to be dried out.

In the DLS measurement of the DWNTs, a spurious peak was found and removed for the calculations. In spite of this, again, the measured value of the mean length is notably smaller than that found by the EB-based methods (Table 1).

### Gibbsite platelets

**Figure 6:**
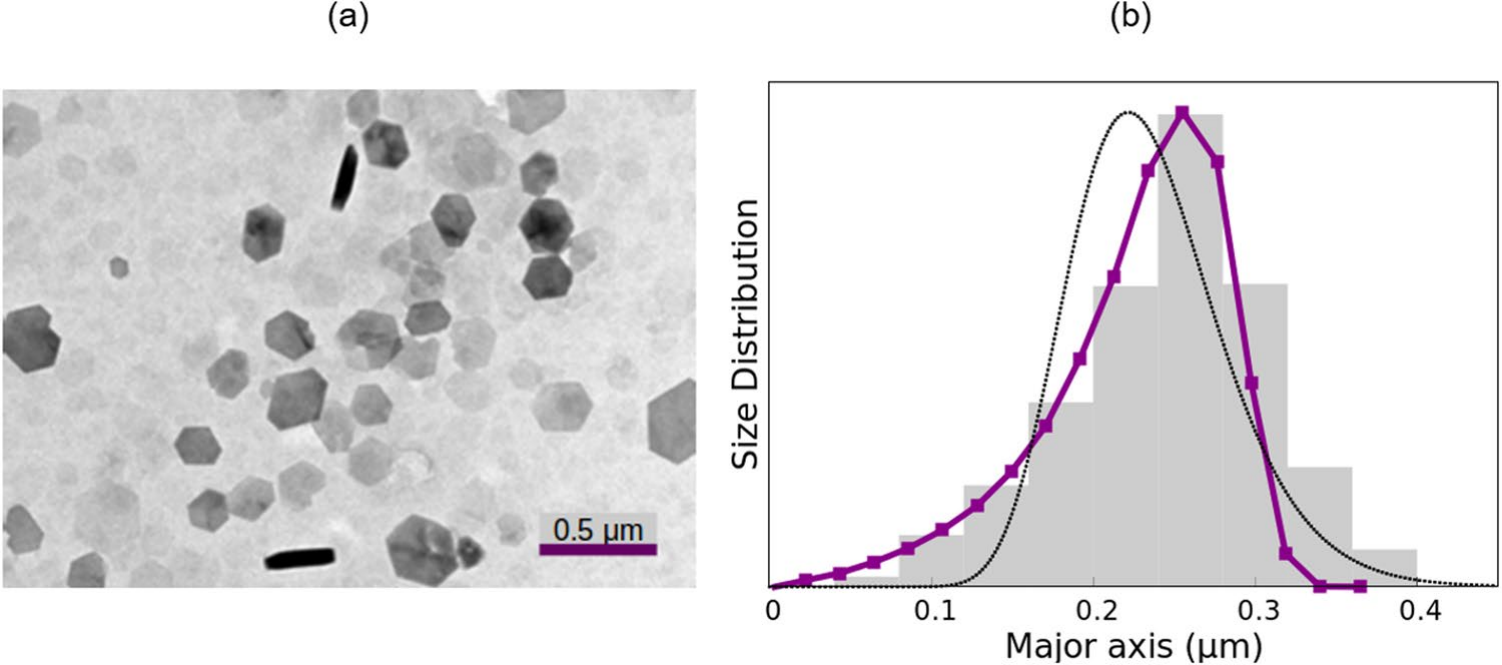
Same as Fig. 2 but for the gibbsite platelets.

We also considered the birefringence decay of gibbsite platelets, studied in aqueous suspension with a particle concentration of 0.1% v/v and 0.1 mM potassium chloride, under a 30 V/mm electric field of 100 kHz. In the transmission electron microscope image of the sample (Fig. 6a) the platelets can be easily told apart. From this picture, a volume-averaged length of 0.25 *μ*m was obtained. Since the particles are very slim, the thin disk approximation was used for the calculations. The results are presented as before in Table 1 and Fig. 6b.

The decay of the birefringence can be suitably fitted to a stretched exponential function (*τ* = 8.7 ms, *α* = 0.501). The SE method provides a mean diameter of 0.23 *μ*m, very similar to the microscopy value and identical to the ME result. The size distribution obtained by the ME method is in very good agreement with the EM determinations, although there is a slight underestimation of the presence of particles over 0.3 *μ*m that accounts for the 8% difference in the mean length values.

In the case of the WJ method, although the obtained average length and standard deviation are similar to the ones given by the other techniques, the distribution is somewhat different, with the peak slightly shifted to shorter sizes. This could be due to the log-normal distribution assumed in this procedure, that does not seem to account for the distribution found by EM.

As for the DLS results, once more, the obtained size distribution is considerably wider and strongly shifted to short lengths as compared to both EM determinations and the SE, ME and WJ results.

### Montmorillonite particles

**Figure 7:**
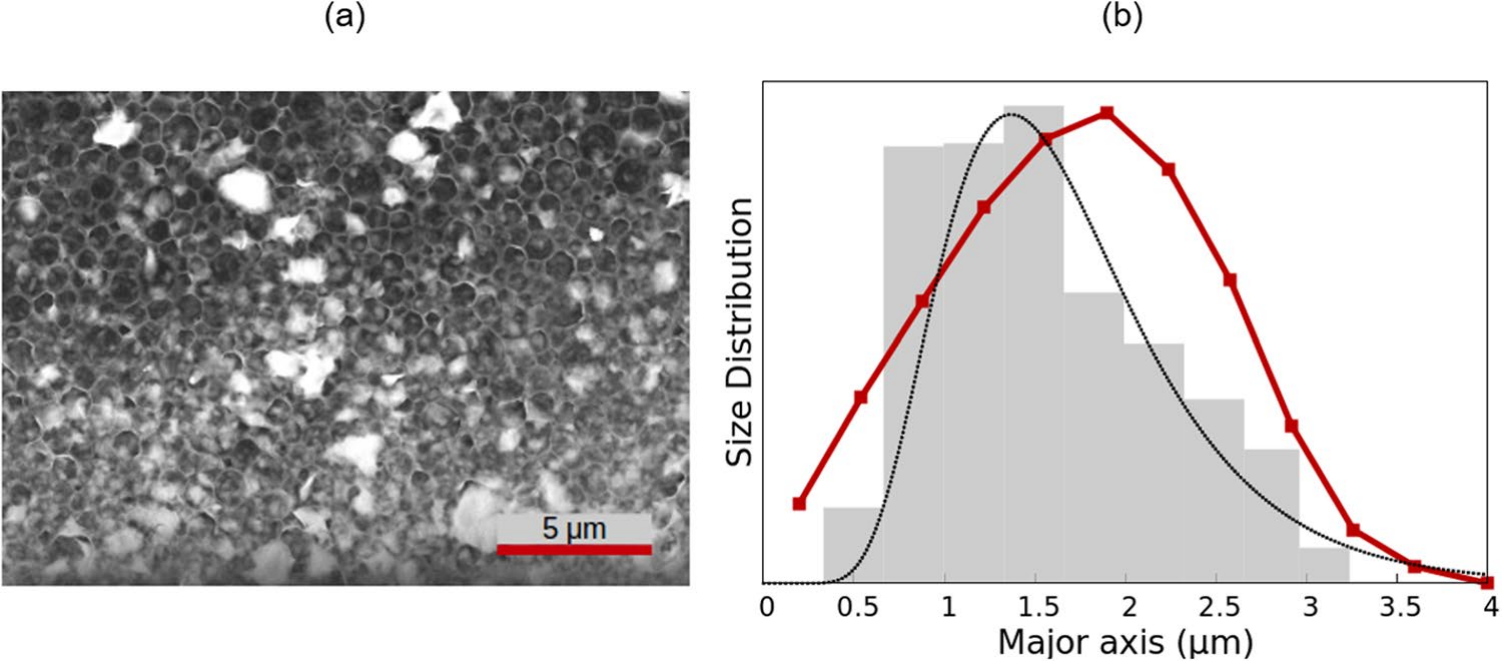
Same as Fig. 2 but for the NaMt sample.

Finally, we analysed the electric birefringence of NaMt particles dispersed in a 0.3 mM sodium chloride solution, with a concentration of 1 g/L. An oscillating electric field of 10 V/mm and a frequency of 1 MHz was applied. An environmental scanning electron microscope picture of the sample is shown in Fig. 7a, where it can be seen that distinction of the particles is quite difficult. From 320 particles, a value of 1.65 *μ*m for the average diameter was obtained. For the calculations, the geometry was modelled by oblate spheroids with a constant aspect ratio of 3.3^46^, an approximation already discussed in the goethite case. The results are presented in Table 1 and Fig. 7b.

The birefringence decay fits perfectly to a stretched exponential function (*τ* = 375 ms, *α* = 0.594), and provides a volume-averaged length of 1.74 *μ*m. This is within 8% of the microscopy value and very similar to the ME result. The latter fails to account for the size distribution found via EM, as it overestimates the presence of long particles. This could be attributed to the approximations used for these particles, which are very irregular and planar, but were approximated by spheroids. In spite of this, both distributions are reasonably similar, with the same standard deviation and a mean length differing only by 7%. The WJ method provides very good results, giving the same value of the mean length as EM and a good shape of the size distribution, although the standard deviation is smaller than measured by microscopy.

For the sodium montmorillonite particles, a spurious peak was found in the DLS measurements and removed for the calculations. The mean length found by this technique is again notably smaller than predicted by all other methods.

## Discussion

In this work, we have compared the performance of three birefringence-based characterization techniques, namely the stretched-exponential, Watson-Jennings and multi-exponential methods, with electron microscopy and DLS measurements. Differently from the other two EB-based approaches, the SE method enables determining only the average size, which we find to match the one obtained by the ME method. This is expected, since the two approaches share common assumptions. The value determined from SE allows a first estimate of the size, which is useful to choose the range of work of the ME method.

The ME method furnishes a complete size distribution of the sample, and the results are excellent in most cases. Thus, for the goethite needles, the SWNTs and the gibbsite platelets, the obtained distributions are in perfect agreement with the microscopy results. For the DWNTs, the ME method gives a length identical to the one found by other techniques^47^ and also by the WJ method, which suggests that these particles are more bundled in suspension than when prepared for microscopy measurements.

For the PTFE and NaMt particles, there are slight differences between the ME and the EM results, but they are not large (below 8%) and can be attributed to the approximations employed such as a constant aspect ratio or the simplified geometry for the rotational diffusion coefficient. Therefore, these differences may be tackled by an improvement of the models. Moreover, distinction of the particles in the microscope pictures is sometimes non-straightforward, as in the case of the SWNTs, which could also account for slight deviations between the results.

The WJ method, in addition to the already mentioned approximations, assumes a fixed shape for the size distribution of the sample, which could be responsible for the larger deviations with respect to the microscopy results that are observed in almost all cases as compared to the ME method. For instance, for the SWNTs, where the sample deviates strongly from the log-normal length distribution, the WJ results differ substantially from the ones obtained by both EM and the ME method.

In our results, it can be observed that the peak of the distributions obtained by the WJ method is systematically shifted to the left as compared with the other procedures. This can be understood taking into account that the log-normal function must go quickly to zero for short lengths, underestimating the presence of small particles, which leads to a shifted maximum. Furthermore, the determination of the initial logarithmic derivative, needed for the WJ calculations, is in some cases non-straightforward, since it is sometimes unclear where the linear behaviour is lost. In spite of this, the results of the WJ method are very satisfactory.

In general, it can be remarked that, except for the DWNTs for the explained reasons, deviations between the mean length obtained by EM and the EB-based methods are in all cases smaller than 8%. This is an excellent result taking into account the very different nature, characteristic sizes and geometries of the selected particles.

On the other hand, the DLS results present strong deviations with respect to the average length found by microscopy, no less than 28% in all cases and up to 74%. Although no systematic behaviour can be confirmed, the DLS distributions present normally a shorter mean length than that found by EM and the SE, ME and WJ methods, which can be attributed to the contribution of rotational diffusion, interpreted as a faster translational diffusion, and hence a smaller size, by the commercial setup. In addition, DLS typically predicts wider standard deviations, a well known feature, analysed elsewhere for elongated geometries^25^. These findings show that the use of commercial DLS setups provides a rough estimation of the characteristic size of non-spherical particles, but not a complete and accurate description of the sample. Nevertheless, these measurements have proven useful, for example, for the characterization of two-dimensional particles, for which a good correlation can be found between the hydrodynamic radii and the dimensions obtained by EM^11^.

In conclusion, the results obtained in this work show that the analysis of transient electric birefringence is a powerful tool for the determination of the size distribution in polydisperse suspensions of non-spherical particles. The multi-exponential method was found to be the most suitable technique for the analysis of the birefringent decay, providing excellent results, although the Watson-Jennings method performance is also very satisfactory.

## Electronic supplementary material

Supplementary Information

## References

[1] Daum N, Tscheka C, Neumeyer A, Schneider M (2012). Novel approaches for drug delivery systems in nanomedicine: effects of particle design and shape. Wiley Interdisciplinary Reviews: Nanomedicine and Nanobiotechnology.

[2] Wen Z, Zhu L, Zhang Z, Ye Z (2015). Fabrication of gas sensor based on mesoporous rhombus-shaped zno rod arrays. Sensors and Actuators B: Chemical.

[3] Ramos-Tejada MM, Espin MJ, Perea R, Delgado AV (2009). Electrorheology of suspensions of elongated goethite particles. journal of non-newtonian fluid mechanics. Journal of Non-Newtonian Fluid Mechanics.

[4] Reiser B, González-García L, Kanelidis I, Maurer J, Kraus T (2016). Gold nanorods with conjugated polymer ligands: sintering-free conductive inks for printed electronics. Chemical Science.

[5] Zhang L (2016). Photodetection and photoswitch based on polarized optical response of macroscopically aligned carbon nanotubes. Nano Letters.

[6] Prodver T (1997). Challenges in particle size distribution measurement past, present and for the 21st century. Progress in organic coatings.

[7] Xu R, Guida OD (2003). Comparison of sizing small particles using different technologies. Powder Technology.

[8] Li M, Wilkinson D (1997). Determination of non-spherical particle size distribution from chord length measurements. part 1: Theoretical analysis. Chemical Engineering Science.

[9] Anderson W, Kozak D, Coleman VA, Jämting Å, Trau M (2013). A comparative study of submicron particle sizing platforms: accuracy, precision and resolution analysis of polydisperse particle size distributions. Journal of Colloid and Interface Science.

[10] Mathaes R, Winter G, Engert J, Besheer A (2013). Application of different analytical methods for the characterization of non-spherical micro-and nanoparticles. International Journal of Pharmaceutics.

[11] Lotya M, Rakovich A, Donegan J, Coleman J (2013). Measuring the lateral size of liquid-exfoliated nanosheets with dynamic light scattering. Nanotechnology.

[12] Levin A, Shmytkova E, Min’kov K (2016). Determination of the geometric parameters of gold nanorods by partially depolarized dynamic light scattering and absorption spectrophotometry. Measurement Techniques.

[13] Li M, Wilkinson D, Patchigolla K (2005). Determination of non-spherical particle size distribution from chord length measurements. part 2: Experimental validation. Chemical Engineering Science.

[14] Krause B, Mende M, Pötschke P, Petzold G (2010). Dispersability and particle size distribution of cnts in an aqueous surfactant dispersion as a function of ultrasonic treatment time. International Journal of Pharmaceutics.

[15] Backes C (2014). Edge and confinement effects allow *in situ* measurement of size and thickness of liquid-exfoliated nanosheets. Nature Communications.

[16] Pecora R (2000). Dynamic light scattering measurement of nanometer particles in liquids. Journal of Nanoparticle Research.

[17] Zhang M, Bradford S, Šimůnek J, Vereecken H, Klumpp E (2017). Roles of cation valance and exchange on the retention and colloid-facilitated transport of functionalized multi-walled carbon nanotubes in a natural soil. Water research.

[18] Reinert L, Zeiger M, Suarez S, Presser V, Mücklich F (2015). Dispersion analysis of carbon nanotubes, carbon onions, and nanodiamonds for their application as reinforcement phase in nickel metal matrix composites. RSC Advances.

[19] Saunders Z, Noack C, Dzombak D, Lowry G (2015). Characterization of engineered alumina nanofibers and their colloidal properties in water. Journal of Nanoparticle Research.

[20] Nelson A, Cosgrove T (2004). Dynamic light scattering studies of poly (ethylene oxide) adsorbed on laponite: Layer conformation and its effect on particle stability. Langmuir.

[21] Kinnear C (2013). Gold nanorods: Controlling their surface chemistry and complete detoxification by a two–step place exchange. Angewandte Chemie International Edition.

[22] Boluk Y, Danumah C (2014). Analysis of cellulose nanocrystal rod lengths by dynamic light scattering and electron microscopy. Journal of Nanoparticle Research.

[23] Liu X (2008). A one-step homogeneous immunoassay for cancer biomarker detection using gold nanoparticle probes coupled with dynamic light scattering. Journal of the American Chemical Society.

[24] Liu T, Xiao Z (2012). Dynamic light scattering of rigid rods–a universal relationship on the apparent diffusion coefficient as revealed by numerical studies and its use for rod length determination. Macromolecular Chemistry and Physics.

[25] Khlebtsov B, Khlebtsov N (2011). On the measurement of gold nanoparticle sizes by the dynamic light scattering method. Colloid Journal.

[26] Levin A, Shmytkova E, Khlebtsov B (2017). Multipolarization dynamic light scattering of nonspherical nanoparticles in solution. The Journal of Physical Chemistry C.

[27] Zimbone M, Messina E, Compagnini G, Fragalà M, Calcagno L (2015). Resonant depolarized dynamic light scattering of silver nanoplatelets. Journal of Nanoparticle Research.

[28] Badaire S, Poulin P, Maugey M, Zakri C (2004). *In situ* measurements of nanotube dimensions in suspensions by depolarized dynamic light scattering. Langmuir.

[29] Bossert D, Natterodt J, Urban D, Weder C, Balog AP-FS (2017). Speckle-visibility spectroscopy of depolarized dynamic light scattering. The Journal of Physical Chemistry B.

[30] Shetty A, Wilkins G, Nanda J, Solomon M (2009). Multiangle depolarized dynamic light scattering of short functionalized single-walled carbon nanotubes. The Journal of Physical Chemistry C.

[31] Martchenko I, Dietsch H, Moitzi C, Schurtenberger P (2011). Hydrodynamic properties of magnetic nanoparticles with tunable shape anisotropy: Prediction and experimental verification. The Journal of Physical Chemistry B.

[32] Lehner D, Lindner H, Glatter O (2000). Determination of the translational and rotational diffusion coefficients of rodlike particles using depolarized dynamic light scattering. Langmuir.

[33] O’Konski, C. *Molecular electro-optics* (Marcel Dekker, New York, 1976).

[34] Frederiq, E. & Houssier, C. *Electric dichroism and electric birefringence* (Clarendon, Oxford, 1973).

[35] Thurston GB, Bowling DI (1969). The frequency dependence of the kerr effect for suspensions of rigid particles. Journal of Colloid and Interface Science.

[36] Holzheu S, Hoffmann H (2002). Mechanistic origin of transient electric birefringence anomaly of clay mineral dispersion. J. Phys. Chem. B.

[37] Bakk A (2002). Viscosity and transient electric birefringence study of clay colloidal aggregation. Phys. Rev. E.

[38] Dozov I (2011). Electric-field-induced perfect anti-nematic order in isotropic aqueous suspensions of a natural beidellite clay. J. Phys. Chem. B.

[39] Petrov MP, Shilov VN, Trusov AA, Voitylov AV, Vojtylov VV (2016). Electro-optic research of polarizability dispersion in aqueous polydisperse suspensions of nanodiamonds. Colloids and Surfaces A: Physicochemical and Engineering Aspects.

[40] Bellini T, Mantegazza F, Piazza R, Degiorgio V (1989). Stretched-exponential relaxation of electric birefringence in a polydisperse colloidal solution. Europhysics Letters.

[41] Degiorgio V, Piazza R, Mantegazza F, Bellini T (1990). Stretched-exponential relaxation of electric birefringence in complex liquids. Journal of Physics: Condensed Matter.

[42] Matsumoto M, Watanabe H, Yoshioka K (1972). A method for determining the relaxation spectrum from the decay curve of electric birefringence of macromolecular solutions. Kolloid-Zeitschrift und Zeitschrift für Polymere.

[43] Watson R, Jennings B (1992). Polydisperse size data from single electric birefrignence transients. Powder Technology.

[44] Wijnhoven J, van’t Zand D, van der Beek D, Lekkerkerker H (2005). Sedimentation and phase transitions of colloidal gibbsite platelets. Langmuir.

[45] Ramos-Tejada M, Arroyo F, Perea R, Durán J (2001). Scaling behavior of the rheological properties of montmorillonite suspensions: correlation between interparticle interaction and degree of flocculation. Journal of Colloid and Interface Science.

[46] Arenas-Guerrero P, Iglesias G, Delgado A, Jiménez M (2016). Electric birefringence spectroscopy of montmorillonite particles. Soft Matter.

[47] Arenas-Guerrer P, Jiménez M, Scott K, Donovan K (2018). Electric birefringence of carbon nanotubes: Single-vs double-walled. Carbon.

