# Supplementary material for: Determination of the size distribution of non-spherical nanoparticles by electric birefringence-based methods
Source: Sci Rep. 2018 Jun 22;8:9502. doi: 10.1038/s41598-018-27840-0 (PMC6015062; doi:10.1038/s41598-018-27840-0)
Supplement: Supplementary file 1 — Supplementary Information [file 41598_2018_27840_MOESM1_ESM.pdf]

Determination of the Size Distribution of Non-Spherical Nanoparticles by Electric Birefringence-Based Methods

Paloma Arenas-Guerrero, Ángel V. Delgado, Kevin J. Donovan, Kenneth Scott, Tommaso Bellini, Francesco Mantegazza, María L. Jiménez*

Supporting information

1 Translational and rotational diffusion coefficients of non-spherical particles

The rotational and translational diffusion coefficients of suspended particles, D and Θ respectively, can be calculated as indicated in Equations 1 and 2 of the main text, where F_D and F_Θ are geometrical factors depending only on the particle shape. In this work, we used the expressions of D and Θ for several simplified geometries, namely, short rods,[1] long rods,[2] thin disks[3] and oblate spheroids.[4] The expressions of F_D and F_Θ corresponding to these particle shapes are presented in Table S1.

Table S1. Expressions of the coefficients F_D and F_Θ in equations 1 and 2 of the main text, for the simplified geometries used in this work.

Short rods	Long rods
$F_D = \log\rho + 0.312 + 0.565/\rho - 0.1/\rho^2$<br>$F_\Theta = \log\rho - 0.662 + 0.918/\rho + 0.05/\rho^2$	$F_D = \log\rho + 0.312 + 0.565/\rho - 0.1/\rho^2$<br>$F_\Theta = \log\rho - 0.2/(\log 2\rho) - 16/(\log 2\rho)^2$
**Thin disks**	**Oblate spheroids**
$F_D = \pi/2$<br>$F_\Theta = \pi/4$	$F_D = \rho \arctan(\sqrt{\rho^2-1})/\sqrt{\rho^2-1}$<br>$F_\Theta = \rho^3((2-\rho^2)G_\Theta - 1)/(2-2\rho^4)$<br>$G_\Theta = \arctan(\sqrt{\rho^2-1})/\sqrt{\rho^2-1}$

2 Experimental birefringence decay curves

The normalised birefringence decays of all the suspensions studied in this work are shown in Fig. S1, where it can be observed that the data are suitably fitted by stretched exponential functions in all cases. In this figure, we also show the decays in logarithmic scale for short times, in order to calculate the initial logarithmic derivative required for the WJ method.

3 Data analysis

The birefringence decay of a monodisperse sample is known to be in the form of a single-exponential function, with characteristic time τ_m:

$$\Delta n(t) = \Delta n_0 \exp[-t/\tau_m] \tag{S1}$$

For a polydisperse system, the overall signal is a superposition of these independent exponential processes, and the contribution of every population must be taken into account. In this manner, the birefringence signal can be expressed as

$$\Delta n(t) = \Delta n_0 \int P(L)S(L)\exp[-t/\tau(L)]dL \tag{S2}$$

where $\tau(L)$ is the decay time for a particle with characteristic dimension L, $P(L)$ is the size distribution of the sample and $S(L)$ measures the contribution of each independent process to the total birefringence signal. It is further assumed that $P(L) \propto \exp(-AL^p)$, $S(L) \propto L^r$ and $\tau(L) \propto L^q$, where A is a constant and p, q and r positive integers. As a result, it has been demonstrated that[5]

$$\Delta n(t) \propto t^\gamma \exp[-t/\tau_m] \tag{S3}$$

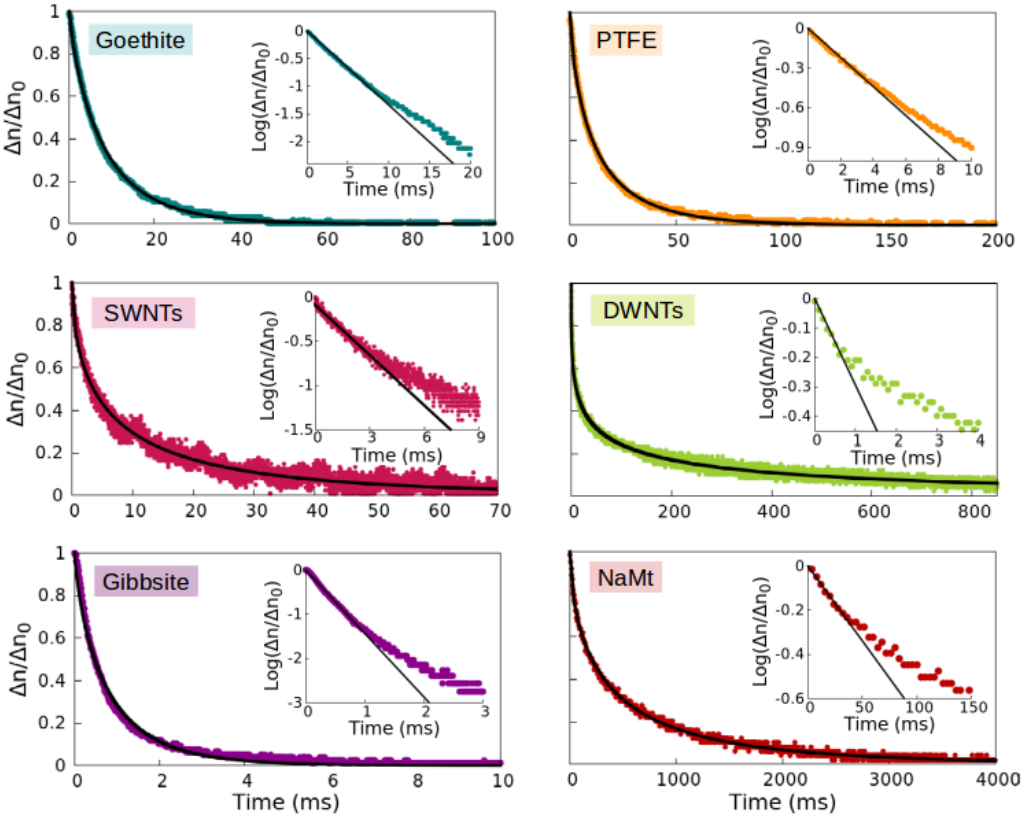

Figure S1. Normalized birefringence decays of the studied suspensions. The points are the experimental data, and the black lines fittings to stretched exponential functions. In the insets, the data for short times (points) are presented in logarithmic scale, along with a linear fitting (black line), to show the linear dependence.

where the contribution of the t^{γ} factor is only significant at very short times, and is neglected to obtain equation 3 of the main text. A non-linear fitting of the birefringence decay data to this stretched-exponential function yields the parameters α and τ, and from the latter the rotational diffusion coefficient is obtained.

A more realistic shape for the size distribution is typically the log-normal function (equation 4 of the main text). Assuming this distribution and calculating the average of the rotational diffusion coefficient of the particles and its inverse, it is possible to relate the distribution parameters, L_M and σ to the initial logarithmic derivative and the area under the curve of the normalised birefringence decay. Details of the derivation of equations 5 and 6 of the main text can be found in the original reference.[3] The area under the curve, I_{WJ}, is calculated numerically, whereas the initial derivative, D_{WJ} is obtained from a linear fitting of the short-time decay data, as shown in Figure S1.

In the multi-exponential method, no assumption is made for the shape of the size distribution of the sample. Instead, a superposition analogous to that made in equation S2 is used in the discrete form, as indicated in equation 7 of the manuscript. For the data analysis, the experimental results are previously fitted to a stretched-exponential function, in other to smooth the time dependence. Then, this function is sampled, and M=10.000 pairs of t_j, $\Delta n(t_j)$ are taken. Later, N size intervals, typically around 10, are chosen. The covered size range must be reasonable, and can be estimated from the SE result. Finally, a linear equation system is obtained, which can be expressed in matrix form as:

$$\begin{pmatrix} \Delta n(t_1) \\ \Delta n(t_2) \\ \dots \\ \Delta n(t_M) \end{pmatrix} = \begin{pmatrix} e^{-t_1/\tau_1} & e^{-t_1/\tau_2} & \dots & e^{-t_1/\tau_N} \\ e^{-t_2/\tau_1} & e^{-t_2/\tau_2} & \dots & e^{-t_2/\tau_N} \\ \dots & \dots & \dots & \dots \\ e^{-t_1/\tau_M} & e^{-t_M/\tau_2} & \dots & e^{-t_M/\tau_N} \end{pmatrix} \begin{pmatrix} C_1 \\ C_2 \\ \dots \\ C_N \end{pmatrix} \tag{S4}$$

Here, the only unknowns are the C_i, obtained numerically by solving the matrix equation with Matlab®.

4 Effect of the constant aspect ratio approximation

For goethite needles, PTFE rods and NaMt particles, the aspect ratio is assumed to be constant in order to carry out the required calculations. As discussed in the main text, this approximation is not expected to have a strong effect on the final results, due to the smooth dependence of the rotational diffusion on ρ, as compared to the major axis length. As an example, Figure S2 shows the size distribution of the goethite sample obtained by the ME method using the constant aspect ratio and the constant particle diameter assumptions. The differences are not very pronounced, indicating that the chosen approximation is not critical. Nevertheless, it is clear that the constant ρ assumption yields better results, since it is more reasonable for this system.

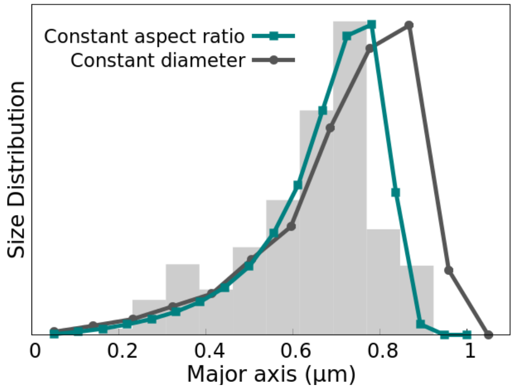

Figure S2. Comparison of the size distributions obtained by the ME method for the goethite sample, assuming a constant aspect ratio and a constant particle width. The microscopy result (grey bars) is also shown.

References

1. Ortega, A. & de la Torre, J. Hydrodynamic properties of rodlike and disklike particles in dilute solution. *J. Chem. Phys.* **119(18)**, 9914–9919 (2003).

2. Tirado, M., Martínez, C. & de la Torre, J. Comparison of theories for the translational and rotational diffusion coefficients of rod-like macromolecules. application to short dna fragments. *J. Chem. Phys.* **81(4)**, 2047–2052 (1984).

3. Watson, R. & Jennings, B. Polydisperse size data from single electric birefrignence transients. *Powder Technol.* **72(1)**, 63–69 (1992).

4. Pecora, R. *Dynamic light scattering: applications of photon correlation spectroscopy* (Plenum Press, New York and London, 1985).

5. Bellini, T., Mantegazza, F., Piazza, R. & Degiorgio, V. Stretched-exponential relaxation of electric birefringence in a polydisperse colloidal solution. *Europhys. Lett.* **10 (5)**, 499–503 (1989).
